# H_2_O_2_-Responsive Hormonal Status Involves Oxidative Burst Signaling and Proline Metabolism in Rapeseed Leaves

**DOI:** 10.3390/antiox11030566

**Published:** 2022-03-16

**Authors:** Bok-Rye Lee, Van Hien La, Sang-Hyun Park, Md Al Mamun, Dong-Won Bae, Tae-Hwan Kim

**Affiliations:** 1 Department of Animal Science, Institute of Agricultural Science and Technology, College of Agriculture & Life Science, Chonnam National University, Gwangju 61186, Korea; turfphy@jnu.ac.kr (B.-R.L.); trungtamnccttubdkh@tuaf.edu.vn (V.H.L.); ghost1284@jnu.ac.kr (S.-H.P.); 187054@jnu.ac.kr (M.A.M.); 2 Department of Biotechnology and Food Technology, Thai Nguyen University of Agriculture and Forestry, Thai Nguyen 24000, Vietnam; 3 Central Instruments Facility, Gyeongsang National University, Jinju 52828, Korea; bdwon@gnu.ac.kr

**Keywords:** abscisic acid, *Brassica napus*, drought, hydrogen peroxide, proline, salicylic acid

## Abstract

Drought alters the level of endogenous reactive oxygen species (ROS) and hormonal status, which are both involved in the regulation of stress responses. To investigate the interplay between ROS and hormones in proline metabolism, rapeseed (*Brassica napus* L.) plants were exposed to drought or exogenous H_2_O_2_ (Exo-H_2_O_2_) treatment for 10 days. During the first 5 days, the enhanced H_2_O_2_ concentrations in drought treatment were associated with the activation of superoxide dismutase (SOD) and NADPH oxidase, with enhanced ABA and SA levels, while that in Exo-H_2_O_2_ treatment was mainly associated with SA-responsive POX. During the latter 5 days, ABA-dependent ROS accumulation was predominant with an upregulated oxidative signal-inducible gene (*OXI1*) and *MAPK6,* leading to the activation of ABA synthesis and the signaling genes (*NCED3* and *MYC2*). During the first 5 days, the enhanced levels of P5C and proline were concomitant with SA-dependent *NDR1*-mediated signaling in both drought and Exo-H_2_O_2_ treatments. In the latter 5 days of drought treatment, a distinct enhancement in *P5CR* and *ProDH* expression led to higher proline accumulation compared to Exo-H_2_O_2_ treatment. These results indicate that SA-mediated P5C synthesis is highly activated under lower endogenous H_2_O_2_ levels, and ABA-mediated *OXI1*-dependent proline accumulation mainly occurs with an increasing ROS level, leading to *ProDH* activation as a hypersensitive response to ROS and proline overproduction under severe stress.

## 1. Introduction

Reactive oxygen species (ROS) are generated due to the univalent reduction of oxygen in the metabolic pathway as one of the earliest responses of plant cells to drought [1,2,3,4,5,6] and pathogen infection [7,8,9]. Excess of ROS causes oxidative stress that can damage proteins, lipids, and DNA [10,11,12]. ROS also function as secondary messengers in the regulation of stress responses in plants [13,14,15]. Thus, the steady-state level of ROS in cells needs to be tightly regulated by ROS-scavenging and ROS-producing proteins, such as peroxidases (POXs), NADPH oxidase, superoxide dismutase (SOD), and catalase (CAT) [16,17,18], as well as by non-enzymatic metabolic pathways (e.g., glutathione-ascorbate cycle) [19,20]. As the most stable among the ROS, H_2_O_2_ is appropriate to play this function [2,17,21]. H_2_O_2_ produced by cytosolic membrane-bound NADPH oxidase is the key player associated with the ROS-related signal transduction [14,20,21]. Oxidative burst-mediated signaling is required for the induction of the *oxidative signal-inducible gene* (*OXI1*). The *OXI1* encoding a serine/threonine kinase is induced in response to a wide range of H_2_O_2_-generating stimuli [13]. Activation of OXI1 results in the activation of a mitogen-activated protein kinase (MAPK) cascade (MAPK3/6) and the induction or activation of different transcription factors that regulate the ROS-scavenging and ROS-producing pathways [2]. In addition, plants exposed to stress stimuli often upregulate ROS (especially H_2_O_2_) and phytohormone signaling [5,8,22]. In this regard, the interaction between H_2_O_2_ and hormones has been widely studied under different environmental stresses in various plants [6,20,23,24].

Another common response to drought stress is the accumulation of proline along with enhanced H_2_O_2_ levels. The H_2_O_2_ produced by NADPH oxidase increases proline accumulation to scavenge ROS [25,26,27], whereas overproduced proline leads to increase in endogenous ROS [28,29,30]. Numerous studies have shown that ROS and proline accumulation are regulated by stress-responsive hormones, of which the best studied are abscisic acid (ABA) and salicylic acid (SA). Drought, in general, increases levels of both endogenous ABA and SA, as well as their signaling along with an enhanced H_2_O_2_ level [5,6,31]. ROS (particularly H_2_O_2_) is thought to be a part of ABA signaling. For instance, drought-enhanced H_2_O_2_ from NADPH oxidase [27,32] induces proline accumulation via upregulation of pyrroline-5-carboxylate synthetase (P5CS) and downregulation of proline dehydrogenase (ProDH) [5,33] in an ABA-dependent manner [31,34]. Our previous studies have shown that severe drought symptoms, characterized by the ABA-responsive proline and H_2_O_2_ accumulation leading to the oxidized state of redox, are alleviated by a SA-mediated antagonistic depression of ABA responses [5,31]. Despite the increasing evidence of a close relationship between ROS and proline metabolism linked to hormonal interaction, the hormonal regulation of proline metabolism in relation to endogenous H_2_O_2_ levels and different H_2_O_2_ sources (e.g., drought-induced and exogenous H_2_O_2_), which are partially associated with the discrepancies observed in their regulatory roles in stress response and resistance processes, has rarely been studied.

In the present study, we hypothesized that (1) the regulatory actions of drought-induced H_2_O_2_ (as the internal H_2_O_2_ trigger) and of exogenous H_2_O_2_ are different in ROS signal transduction and (2) the altered H_2_O_2_ levels and their signaling modulate proline metabolism with hormonal interaction. To test these hypotheses, antioxidant activity, ABA and SA responses, and proline metabolism were interpreted with respect to the altered H_2_O_2_ levels and ROS signaling, in response to drought or exogenous-H_2_O_2_ treatment.

## 2. Materials and Methods

### 2.1. Plant Materials, Growth Conditions, and Stress Treatments

Plants of the rapeseed (*Brassica napus* L.) cultivar Capitol were used for this study (Gwangju, Korea) and cultivated as previously described by Lee. et al. [35]. The seedlings at four-leaf stage were transferred to soil-filled 2 L pots and irrigated continuously in complete nutrient solution for 6 weeks. Then, plants were divided into three groups according to morphological similarity. The first group was irrigated with 200 mL of water for the well-watered plants (control), the second with 20 mL of water (drought), and the third was daily foliar-sprayed with 20 mL of 50 µM H_2_O_2_ under well-watered conditions (exogenous-H_2_O_2_ (Exo-H_2_O_2_)) for 10 days. Sampling was performed at 0, 5, and 10 days after treatment. In this study, the mature leaves ranked 4–12 (i.e., rank 1 for the oldest leaf) were considered. After sampling, leaf tissues were cut and frozen immediately in liquid nitrogen and stored in a deep-freezer (−80 °C) until further analysis.

### 2.2. Measurement of Leaf Water Potential (Ψ_w_) and Chlorophyll Content

For measurement of leaf water potential (*Ψ_w_*), the seventh leaf was cut and then inserted the pressure chamber (PMS Instruments, Corvallis, OR, USA) to expose the cut end of the petiole on the outside. Afterwards, pressure was applied to the chamber until liquid was observed at the end of petiole, which corresponds to the *Ψ_w_*. For total chlorophyll, approximately 100 mg of fresh-cut leaves were extracted with 10 mL of 99% dimethyl sulfoxide [36]. After 48 h, the absorbance of the supernatants was read at 645 and 663 nm and calculated using the following formula: total chlorophyll (µg/mL) = 20.2 A_645_ + 8.02 A_663_.

### 2.3. Dtermination of Phytohormones

For the quantification of phytohormones, 50 mg of finely ground fresh leaves was extracted with 500 µL of the extraction solvent (2-propanol/H_2_O/concentrated HCl (2:1:0.002, *v*/*v*/*v*)) containing d_6_-ABA and d_6_-SA as the internal standard (50 ng) for ABA and SA, respectively, for 24 h at 4 °C [37]. The supernatant was mixed with 1 mL of dichloromethane and at 13,000× *g* for 5 min at 4 °C. After centrifugation, two phases were formed. The supernatant in the lower phase was transferred to clean screw-cap glass vial and dried using a nitrogen evaporator with nitrogen flow. Then, samples were re-suspended in 1 mL of methanol and further purified with filtering through 0.22 μm organic membrane filters. The extracted solution transferred to vials with a glass insert and stored at −80 °C until high-performance liquid chromatography electrospray ionization tandem mass spectrometry (HPLC-ESI-MS/MS) analysis. Ten microliters of plant extracts were injected onto an Agilent 1100 HPLC system, equipped with a Waters C18 column (150 × 2.1 mm, 5µm) and API3000 MS-MRM (Applied Biosystems, Waltham, MA, USA).

### 2.4. Antioxidant Enzyme Activities

For extraction of antioxidant enzymes, approximately 500 mg of finely ground fresh samples were extracted with 1.5 mL of 100 mM potassium phosphate buffer pH 7.5 containing 2 mM phenylmethylsulfonyl fluoride. After centrifugation at 14,000× *g* for 20 min at 4 °C, the supernatants were used as enzyme sources [38]. Protein concentration was determined using Bradford reagent with bovine serum albumin as a proteins standard. For cell wall POX activity, the oxidation of guaiacol was evaluated by monitoring the increase in absorbance at 470 nm for 1 min (coefficient of absorbance, ε = 26.6 mM^−1^ cm^−1^) [39]. One unit of enzyme activity was defined as the amount of enzyme causing the formation of 1 M tetraguaiacol per min. SOD activity was measured by its ability to inhibit the photoreduction of nitroblue tetrazolium (NBT) [11]. One unit of enzyme activity was defined as the amount of enzyme causing 50% inhibition of NBT photoreduction in comparison with tubes lacking the plant extract. CAT activity was monitored by following the decrease in absorbance at 240 nm due to H_2_O_2_ consumption (ε = 36 mM^−1^ cm^−1^). One unit of enzyme activity was defined as the amount of enzyme causing the degradation of 1 µmol of H_2_O_2_ per min [38].

### 2.5. Chemical Analysis

Fresh samples (0.5 g) were mixed with 1.5 mL of 50 mM KPO_4_^−^ buffer (pH 7.0) and centrifuged at 10,000× *g* for 25 min at 4 °C. After centrifugation, the supernatants were used to determine the superoxide anion radical (O_2_^•–^) and H_2_O_2_ concentration. The O_2_^•–^ concentration was conducted according to the method of Lee et al. [11] by O_2_^•–^ oxidation of hydroxylamine. Briefly, the supernatants were mixed with hydroxylamine solution, incubated for 1 h at 25 °C, and then reacted with 17 mM sulfanilic acid and 7 mM a-naphthylamine for 20 min. The absorbance was read at 530 nm and calculated using the NO_2_ standard. The H_2_O_2_ concentration was measured by the method of Lin and Kao [40]. The supernatants were mixed with 0.1% titanium chloride in 20% H_2_O_2_. The absorbance was immediately read at 410 nm. The H_2_O_2_ concentration was determined by measuring the absorbance at 410 nm and calculated using the extinction coefficient of 0.28 mM^−1^ cm^−1^. The lipid peroxidation level was determined by measuring the concentration of malondialdehyde (MDA), as described previously [3]. Fresh samples (0.5 g) were extracted with 0.1% trichloroacetic acid, centrifuged, mixed with 0.5% tribabutric acid in 20% TCA, and then boiled for 30 min at 95 °C. The absorbance was measured at 532 nm and calculated using the extinction coefficient of 155 mM^−1^ cm^−1^. The concentration of proline and pyrroline-5-carboxylate (P5C) was measured using the method described by Bates et al. [41] and Deuschle et al. [42], respectively. Proline and P5C contents in the eluate were quantified using ninhydrin assay and the *o*-aminobenzaldehyde method, respectively.

### 2.6. RNA Isolation and Expression Quantification

Fresh leaves (200 mg) were mixed with RNAiso Plus reagent (Takara, Nojihigashi 7-4-38 Kusatsu, Shiga, Japan) for total RNA isolation. First-strand complementary DNA (cDNA) was synthesized with a GoScript Reverse Transcription System (Takara). RT-qPCR reactions were carried out on a BioRad CFX96 qPCR System using the TB Green Premix Ex Taq (Takara). Three biological replications were carried out for each treatment, each with two technical replicates. The relative expression levels of were normalized to actin and calculated using the 2^−^^ΔΔ^^Ct^ method [43]. Supplementary Table S1 provides the gene-specific primer used for qRT-PCR.

### 2.7. Statistical Analysis

All measurements were performed with three replicates per treatment. The experimental results are presented as mean ± SE. Duncan’s multiple range test was performed to compare the means of separate replicates. Statistical significance was established at *p* < 0.05. Statistical analysis of all measurements was performed using SAS 9.1.3 software (SAS Institute Inc., Cary, NC, USA). Heatmap and Pearson correlation analyses were conducted using MetaboAnalyst 4.0 (http://www.metaboanalyst.ca, assessed on 20 September 2021).

## 3. Results

### 3.1. Leaf Water Potential, Chlorophyll Content, and Lipid Peroxidation Level

Drought and Exo-H_2_O_2_ treatments for 10 days significantly decreased the leaf water potential (*Ψ_w_*) (Table 1). The drought-responsive decrease in *Ψ_w_* was earlier and higher than that observed in Exo-H_2_O_2_ treatment. The chlorophyll content on day 10 also tended to decrease in both drought and Exo-H_2_O_2_ treatments compared to the control (Table 1). The concentration of MDA, as a marker of lipid peroxidation caused by oxidative stress, significantly increased to 2.0- and 1.3-fold in drought and Exo-H_2_O_2_ treatments, respectively, compared to the control on day 10 (Table 1).

### 3.2. ROS Status and Antioxidative Enzymes Activity

**Figure 1:**
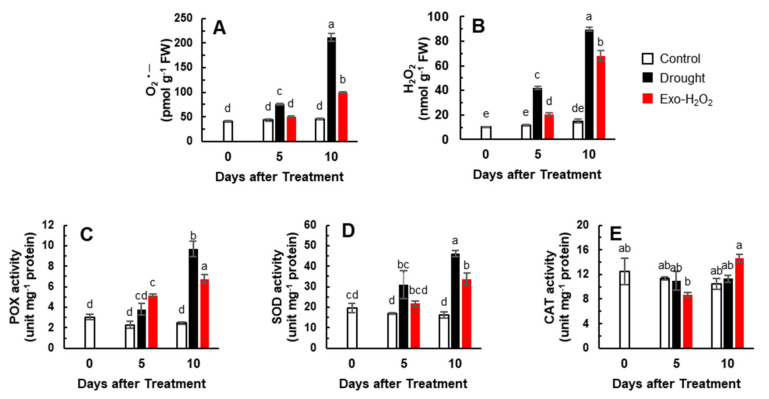
Changes in the concentration of ROS and in the activity of antioxidative enzymes in the leaves of control, drought−, or exogenous H_2_O_2_ (Exo−H_2_O_2_) −treated plants for 10 days. (**A**) O_2_^•–^ and (**B**) H_2_O_2_ concentration, (**C**) peroxidase (POX), (**D**) superoxide dismutase (SOD), and (**E**) catalase (CAT) activity. Results are represented as mean ± SE for *n* = 3. Different letters indicate values that are significantly different at *p* < 0.05 according to Duncan’s multiple range test.

To quantify the oxidative responses of plants to drought or Exo-H_2_O_2_ treatment, we determined the O_2_^•–^ and H_2_O_2_ contents and enzyme activities of POX, SOD, and CAT. The O_2_^•–^ concentration significantly increased in both the treatments throughout the experimental period (except on day 5 of Exo-H_2_O_2_ treatment). On day 10, the accumulation of O_2_^•–^ was much higher in drought treatment as it showed a 5.2-fold increase, while Exo-H_2_O_2_ treatment showed a 2.4-fold increase when compared to the control on day 0 (40.6 pmol g^−1^ FW) (Figure 1A). A significant increase in H_2_O_2_ concentration and accumulation was also observed in both treatments. Drought-responsive H_2_O_2_ accumulation was higher than that in Exo-H_2_O_2_ treatment (Figure 1B). The enzymatic activity of cell wall POX also increased in both the treatments. The activation of POX in both drought and Exo-H_2_O_2_ treatments was not significant on day 5, whereas it was 1.4-fold higher in drought treatment than that in Exo-H_2_O_2_ treatment on day 10 (Figure 1C). The activity of SOD progressively increased with time in both the treatments. The SOD activity on day 10 was 46.2 and 33.8 unit mg^−1^ protein in drought and Exo-H_2_O_2_ treatments, respectively (Figure 1D). The CAT activity was not significantly different between treatments throughout the experimental period (Figure 1E). These results indicated that the endogenous H_2_O_2_ level affects the ROS status and activities of antioxidant enzymes.

### 3.3. Endogenous ABA and SA Status, and ABA and SA Synthesis and Signaling Genes Expression

In order to effects of drought or Exo-H_2_O_2_ treatment on phytohormone metabolism, ABA and SA contents and their synthesis- and signaling-related gene expressions were measured. Endogenous ABA level on day 5 was significantly increased only in drought-treated leaves. The accumulation of ABA occurred in both treatments, with 22- and 6.4-fold increase in drought and Exo-H_2_O_2_ treatments, respectively, compared to the control on day 0 (Table 2). Levels of SA were significantly higher on day 5, then decreased on day 10 in both treatments. The highest SA level was recorded on day 5 in the Exo-H_2_O_2_ treatment, with a 1.9-fold higher level than that measured in the drought treatment (Table 2). The resulting ratio of ABA/SA on day 10 was 27.55 and 6.12 in drought and Exo-H_2_O_2_ treatments, respectively (Table 2).

**Figure 2:**
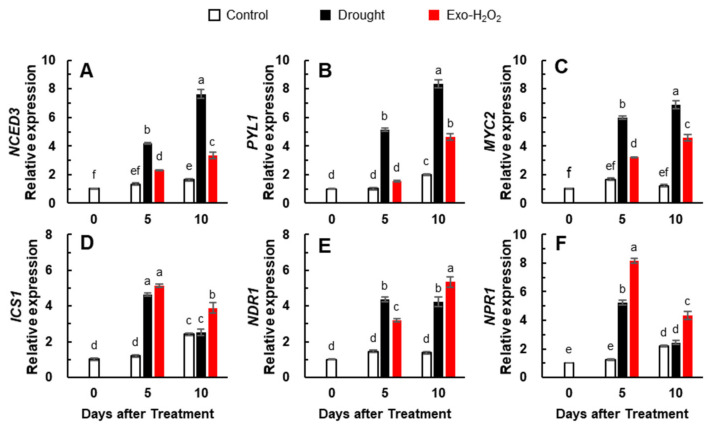
Changes in the expression of (**A**) ABA synthesis-related gene *NCED3*, (**B**) ABA receptor gene *PYL1*, (**C**) ABA signaling-related gene *MYC2*, (**D**) SA synthesis-related gene *ICS1*, (**E**) *NDR1*, and (**F**) SA signaling-related gene *NPR1* in the leaves of control, drought-, or exogenous H_2_O_2_ (Exo-H_2_O_2_)-treated plants for 10 days. Results are represented as mean ± SE for *n* = 3. Different letters indicate values that are significantly different at *p* < 0.05 according to Duncan’s multiple range test.

The expression of the ABA synthesis-related gene, *9-cis-epoxycarotenoid dioxygenase* (*NCED3*), was enhanced from day 5 (e.g., 4.2- and 2.3-fold in drought and Exo-H_2_O_2_ treatments, respectively) and continued until day 10. The enhancement of *NCED3* was much higher in the drought treatment (7.6-fold) (Figure 2A). The expressions of the ABA receptor gene (*PYL1*) and ABA signaling gene (*MYC2*) were also highly enhanced in the drought treatment compared to that in Exo-H_2_O_2_ treatment throughout the experimental period (Figure 2B,C). Higher expression of the SA synthesis gene, *iso-chorismate synthase 1* (*ICS1*), was observed in both drought (4.6-fold) and Exo-H_2_O_2_ treatments (5.1-fold) on day 5, which then decreased up until 10 days, but the expression of *ICS1* was significantly higher in Exo-H_2_O_2_ than in the drought treatment (Figure 2D). The expression of the *non-race-specific-disease resistance 1 gene (NDR1*) on day 5 was significantly higher in drought treatment than in Exo-H_2_O_2_ treatment, but it reversed on day 10 (Figure 2E). The expression of the SA signaling gene, *non-expressor of pathogenesis-related gene* (*NPR1*), followed a similar pattern of endogenous SA level throughout the experimental period (Figure 2F). Therefore, it appears that the SA synthesis- and signaling-related pathway is promoted at a lower endogenous H_2_O_2_ level, whereas the ABA synthesis- and signaling-related pathway is enhanced at a higher endogenous H_2_O_2_ level.

### 3.4. Production of ROS and Expression of ROS Signaling Genes

**Figure 3:**
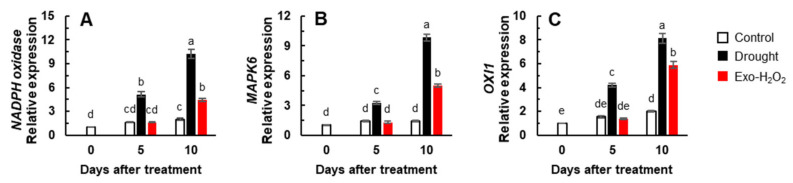
Changes in the expression of (**A**) *NADPH oxidase*, (**B**) transcription factor *MAPK6*, and (**C**) *oxidative signal-inducible* (*OXI1*) gene in the leaves of control, drought-, or exogenous H_2_O_2_ (Exo-H_2_O_2_)-treated plants for 10 days. Results are represented as mean ± SE for *n* = 3. Different letters indicate values that are significantly different at *p* < 0.05 according to Duncan’s multiple range test.

The expression of *NADPH oxidase*-encoding gene was significantly upregulated only in the drought treatment (5.1-fold) on day 5, but then was continuously enhanced in both treatments. The enhancement was much higher in drought treatment on day 10, as shown by a 10.2- and 4.4-fold increase in the drought and Exo-H_2_O_2_ treatments, respectively (Figure 3A). One of the ROS-responsive signaling genes, *MAPK6*, was significantly enhanced only in the drought treatment (3.2-fold) on day 5, and highly enhanced in both treatments on day 10 (9.8- and 5.0-fold in drought and Exo-H_2_O_2_ treatments, respectively) (Figure 3B). The expression of *OXI1* showed responses similar to those of *MAPK6*, representing a 8.1- and 5.9-fold higher expression in drought and Exo-H_2_O_2_ treatments, respectively, on day 10 (Figure 3C).

### 3.5. Proline and P5C Concentration, and Proline Metabolism-Related Gene Expression

**Figure 4:**
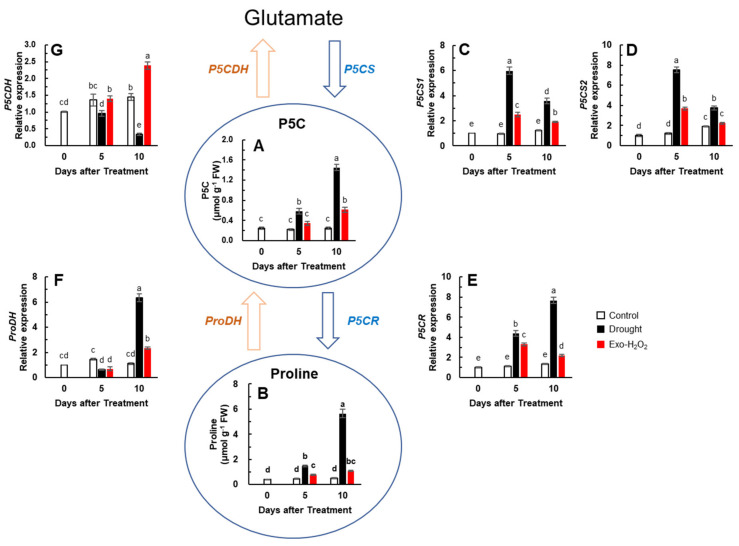
Changes in proline metabolism in the leaves of control, drought-, or exogenous H_2_O_2_ (Exo-H_2_O_2_)-treated plants for 10 days. (**A**) Pyrroline-5-carboxylate (P5C) and (**B**) proline content, and expression of (**C**) *P5C synthase 1* (*P5CS1*), (**D**) *P5CS2*, (**E**) *P5C reductase* (*P5CR*), (**F**) *proline dehydrogenase* (*ProDH*), and (**G**) *P5C dehydrogenase* (*P5CDH*). Results are represented as mean ± SE for *n* = 3. Different letters indicate values that are significantly different at *p* < 0.05 according to Duncan’s multiple range test.

To evaluate the effects of altered H_2_O_2_ levels on proline metabolism, P5C and proline concentrations and proline metabolism-related gene expression were evaluated. Drought Exo-H_2_O_2_ treatments showed a progressive increase in P5C concentration. Drought-responsive enhancement of P5C was significantly higher than Exo-H_2_O_2_-responsive enhancement, as shown by 5.8- and 2.4-fold increased levels after 10 days in drought and Exo-H_2_O_2_ treatments, respectively, compared to the control (Figure 4A). The increase in proline concentration and its accumulation was also significant in both the treatments. The increase in proline concentration was much higher in drought treatment than in Exo-H_2_O_2_ treatment, with a 14.1- and 2.7-fold increase on day 10 in drought and Exo-H_2_O_2_ treatments, respectively, compared to the control on day 0 (Figure 4B). The resulting ratio of proline/P5C on day 10 was 3.9 and 1.8 in drought and Exo-H_2_O_2_ treatments, respectively.

The expression of P5CS-encoding genes (*P5CS1* and *P5CS2*) enhanced in a pattern with a much higher expression in drought treatment during the first 5 days and then a significant decrease in both treatments on day 10 (Figure 4C,D). The expression of P5C reductase-encoding gene (*P5CR*) enhanced to 4.4- and 3.3-fold in drought and Exo-H_2_O_2_ treatments, respectively, on day 5. The *P5CR* expression in drought continued to enhance (7.7-fold), while it remained restricted in Exo-H_2_O_2_ (2.2-fold) treatment (Figure 4E). The expression of *ProDH* reduced in both treatments on day 5, while it was enhanced strongly in drought (6.4-fold) and slightly in Exo-H_2_O_2_ (2.4-fold) treatments on day 10 (Figure 4F). The expression of the P5C dehydrogenase-encoding gene (*P5CDH*) was continuously reduced along with the progression of drought, whereas it was remarkably enhanced on 10 days after Exo-H_2_O_2_ treatment (Figure 4G). Thus, drought-induced H_2_O_2_ leads to high upregulation of P5CR and ProDH expression, resulting in increase of the proline/P5C ratio, compared to Exo-H_2_O_2_ treatment.

### 3.6. Heatmap Visualization and Pearson Correlation Analysis among the Metabolites or Gene Expression

**Figure 5:**
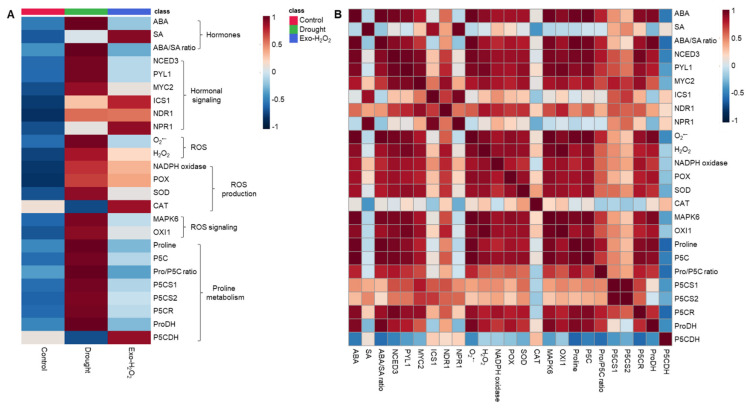
Heatmap analysis of the treatment effect and correlations among the variables measured for 10 days. (**A**) Heatmap comparing the changes of the identified metabolites or gene expression levels in the leaves of control, drought-, or exogenous H_2_O_2_ (Exo-H_2_O_2_)-treated plants for 10 days. The normalization procedure consisted of mean row centering with color scales. (**B**) Heatmap showing the correlations among the identified metabolites or gene expression levels. Correlation coefficients were calculated based on Pearson’s correlation. Red indicates a positive effect, whereas blue indicates a negative effect. Color intensity is proportional to the correlation coefficients.

To further examine the functional implications and correlations of the measured metabolites and gene expression levels in drought and Exo-H_2_O_2_ treatments, a heatmap and Pearson’s correlation coefficients were adapted (Figure 5). Drought had a more positive influence on ABA, ROS, P5C, and proline levels, as well as on the ABA/SA ratio, when compared with that of Exo-H_2_O_2_ (Figure 5A). Altered ROS level, which positively regulated the expression of *MAPK6* and *OXI1*, was closely correlated with ABA and the ABA/SA ratio. The close relationships between ROS and ABA were also found to be positively correlated with P5C and proline, which had a strong correlation with *P5CR* and *ProDH,* as well as with ABA-regulated genes (*NCED3*, *PYL1,* and *MYC2*) (Figure 5B).

## 4. Discussion

### 4.1. ROS and Hormone Responses to Drought-Induced H_2_O_2_ and Exogenous H_2_O_2_

Droughts reduce leaf water potential, which is used as an index of the water statues [3,35]. In the present study, a significant decrease of leaf water potential was observed in drought-stressed plants, accompanied by the loss of chlorophyll and enhanced lipid peroxidation level (Table 1). The accumulation of O_2_^•–^ and H_2_O_2_, an early response to various stress stimuli [6,20], occurred in both drought and Exo-H_2_O_2_ treatments. The endogenous H_2_O_2_ levels in Exo-H_2_O_2_ (68.2 nmol g^−1^ FW) on day 10 corresponded to that of 76% of drought treatment (Figure 1B), which is similar to the results obtained on day 5 of drought treatment in a previous study [6]. During the first 5 days of treatments, the increase in H_2_O_2_ concentration was concomitant with the enhanced SOD activity (Figure 1D) and *NADPH oxidase* expression (Figure 3A) in drought treatment and with POX activity in Exo-H_2_O_2_ treatment (Figure 1C). Wang et al. [44] reported that endogenous H_2_O_2_ accumulation in the apoplast was triggered by both cell wall peroxidase and membrane-linked NADPH oxidase. It has been documented that SA is involved in the production of H_2_O_2_, leading to SA-induced abiotic and biotic stress resistance [20,45]. In the present study, during the first 5 days when the increase in SA levels was predominant, the increased endogenous H_2_O_2_ level coincided with the increased activation of SA-dependent POX in the Exo-H_2_O_2_ treatment, and was mainly due to ABA- and/or SA-dependent SOD activity and NADPH oxidase expression in the drought treatment (Table 2, Figure 1C,D and Figure 3A), in accordance with SA-mediated H_2_O_2_ production by activating membrane-linked NADPH oxidase [46] and cell wall peroxidase [44,47]. During the latter 5 days, when ABA accumulation with an antagonistic depression of SA was remarked, the accumulation of H_2_O_2_ occurred with a proportional enhancement of POX and SOD activity and NADPH oxidase expression in an ABA-dependent manner (Table 2, Figure 1C,D and Figure 3A). Ample evidence has shown that H_2_O_2_ generated by SA-mediated POX and NADPH oxidases acts downstream of ABA signaling in mediating drought-induced stress responses [47,48,49,50]. Our recent study reported that SA-stimulated H_2_O_2_ accumulation and SA responses during the early drought phase are part of upstream H_2_O_2_-stimulated ABA accumulation, which causes ABA signaling and responses, leading to severe drought symptoms during the late phase [6]. The present data indicate that during the first 5 days, H_2_O_2_ is produced mainly from SA-mediated activation of NADPH oxidase in drought treatment and POX in the Exo-H_2_O_2_ treatment, whereas ROS accumulation at day 10 was due to the increase in SOD activity (an H_2_O_2_-producing enzyme), and in NADPH induction (superoxide-producing enzyme), respectively, in an ABA-dependent manner.

### 4.2. H_2_O_2_-Responsive Interaction between ROS and Hormonal Signaling

The altered endogenous H_2_O_2_ level was strongly correlated (*p* < 0.001) to the expression of two protein kinases (*MAPK6* and *OXI1*), which are an essential part of the signal transduction pathway linking oxidative burst signaling to diverse downstream responses [2,13,34,51]. MAPKs, which are downstream of OXI1 [13], are known to be involved in H_2_O_2_ signaling ability for regulating hormonal and metabolic responses [6,22,29,50]. With respect to ROS signaling, a preponderance of evidence supports ABA as a key regulator of stress responses [5,6,34]. Indeed, in the present study, the endogenous ABA level (Table 2) and the expression of *NCED3* and *MYC2* (Figure 2A,C) in drought treatment were consistent with a progressive increase in the endogenous H_2_O_2_ level, leading to a proportional upregulation of *MAPK6* (Figure 3B) and *OXI1* (Figure 3C), while the expression of these genes was not significantly activated in Exo-H_2_O_2_ treatment during the first 5 days when the ABA level did not change significantly (Figure 3). Moreover, the overall pattern of *MAPK6* and *OXI1* (Figure 3B,C) indicated that they are ROS level-responsive (Figure 1A,B) ABA-regulated genes (Figure 2A–C), in accordance with our previous results obtained from a time course of drought intensity [6]. Similarly, in Arabidopsis, overexpression of AtMPK6 enhanced the ABA-dependent H_2_O_2_ production, which is blocked in the *mpk6* mutant [52,53]. In addition, the inhibition of MAPK signaling by PD98059 decreases sensitivity to the response of ABA under drought conditions [54]. The endogenous SA level and the expression of SA-related genes (*ICS1* and *NPR1*) in drought treatment (Figure 2D,F) progressively decreased with an antagonistic increase in the ABA level (Table 2) and ABA-related gene expression (Figure 2A–C), along with ROS accumulation (Figure 1A,B). The SA-signaling genes (*NDR1* and *NPR1*) were highly developed in Exo-H_2_O_2_ on day 5 (Figure 2E,F), in which the endogenous H_2_O_2_ level was relatively lower (≤20 nmol g^−1^ FW) (Figure 1B). These enhanced SA-signaling genes did not significantly activate *MAPK6* and *OXI1* (Figure 3B,C). The time-course analysis showed that the crosstalk between H_2_O_2_ and SA has a much earlier peak than in ABA [6,55]. In the present study, an altered endogenous H_2_O_2_ level was highly correlated with increased ABA and ABA-regulated genes, but not with SA responses (Figure 5B). Therefore, a positive feedback loop between H_2_O_2_- and ABA-mediated pathways might lead to upregulation of *MAPK6* via *OXI1*, thereby activating ABA synthesis and signaling genes (*NCED3* and *MYC2*). Thus, the actions of this core pathway in the control of proline metabolism under drought stress needs to be discussed further.

### 4.3. H_2_O_2_-Responsive Hormonal Regulation of Proline Metabolism

Along with ROS accumulation, proline is the most common free amino acid to accumulate in the plants exposed to drought stress [5,30,35,56]. Based on the data obtained during the entire experimental period, the correlation between endogenous H_2_O_2_ level, P5C, and proline concentration was highly positive, and these parameters were also positively correlated with ROS- and ABA-signaling genes’ expression (Figure 5B). Indeed, it has been documented that H_2_O_2_ causes proline accumulation or promotes ABA-induced proline accumulation [23,57]. Previous results reveal that H_2_O_2_ produced by NADPH oxidase increases proline accumulation under drought or osmotic stress [5,27], with a strong correlation with ABA accumulation [34,56]. However, in the present study, the data collected in Exo-H_2_O_2_, especially during the first 5 days, did not directly match these linear relationships. For instance, the enhanced H_2_O_2_ level in Exo-H_2_O_2_ treatment led to a significant increase in P5C and proline levels (Figure 4A,B), although the ABA level and *NADPH oxidase* gene expression did not change during the first 5 days (Table 2, Figure 3A). The Exo-H_2_O_2_-responsive increases in P5C and proline levels were found to be associated with SA-dependent proline synthesis-related genes (Figure 4C–E), possibly related to SA-mediated activation of POX and not with the NADPH oxidase-dependent process (Figure 1C). Apart from these early responses to Exo-H_2_O_2_, the enhanced P5C and proline levels and their accumulation under drought stress were found to be parallel with upregulated *NADPH oxidase* (Figure 3A) in an ABA-related pattern, with an increasing endogenous-H_2_O_2_ concentration (Figure 1B). These results clearly indicate that drought-induced H_2_O_2_ (often considered the internal H_2_O_2_ trigger) might be directly involved in triggering the NADPH oxidase-dependent proline synthesis, but not in Exo-H_2_O_2_. In drought treatment, P5C and proline levels increased (Figure 4A,B) along with H_2_O_2_ accumulation, with proportional enhancements of ROS-signaling genes (*OXI1* and *MAPK6*) (Figure 3B,C) and ABA-regulated genes (*NCED3, PYL1*, and *MYC2*) (Figure 2A–C), suggesting a greater ABA-dependent proline accumulation in higher H_2_O_2_ level. This observation further indicates that higher proline accumulation does not directly contribute to the scavenging of cellular ROS, although studies show that proline metabolism has a function as ROS scavenger [30]. The proline levels in plant cells depend on tight regulation of its biosynthesis and degradation catabolism. Proline accumulation under stress is accompanied by the upregulation of proline biosynthesis (*P5CS* and *P5CR*) and downregulation of proline catabolism-related genes (*ProDH* and *P5CDH*) [5,6,33,35], in an ABA-dependent manner [31,34]. Indeed, in drought treatment, P5C and proline accumulation occurred with highly enhanced expression of *P5CS1*, *P5CS1*, and *P5CR* (Figure 4C–E), accompanied with a progressive enhancement of *NADPH oxidase* (Figure 3A), which coincided with the enhanced H_2_O_2_-responsive increases in ABA level (Table 2) and ABA-regulated genes’ expressions (Figure 2A–C). This observation confirmed the given hypothesis that H_2_O_2_ generated by NADPH oxidases acts downstream of ABA signaling [48,49,58], and involves in proline accumulation by upregulating *P5CS* [27]. However, in Exo-H_2_O_2_ treatment, the pattern of P5C and proline was not directly associated with those of H_2_O_2_- and ABA-mediated NADPH oxidase, as shown lower activation of *P5CS, P5CR* (Figure 4C–E), and *NADPH oxidase* (Figure 3A), even though endogenous H_2_O_2_ was enhanced up to 68.2 nmol g^−1^ FW on day 10 (Figure 1B). Besides the interaction between H_2_O_2_- and ABA-signaling in proline biosynthesis, it was noteworthy that significant enhancements of *P5CS* and *P5CR* also coincided with the increased SA level and upregulation of SA-related genes (Figure 2D–F), especially when endogenous H_2_O_2_ was less than 42 nmol g^−1^ FW (e.g., during the first 5 days after both treatments). This observation suggests that SA signaling is also involved in the activation of proline synthesis as part of early stress response, confirming the role of SA as a signal of different types of stresses [20,45,59]. In our previous studies, leaf spraying with 30 mL of 0.5 mM SA enhanced enzymatic activity of SOD and their encoding genes, and induced proline accumulation with enhanced synthesis-related genes (*P5CS* and *P5CR*) [5].

Proline is oxidized to glutamate by the sequential action of ProDH and P5CDH [30,60]. In the present study, the expression of *ProDH* tended to decrease with increasing endogenous proline levels, except in the data of 10 days after drought treatment (Figure 4B,F). The enhanced activity of ProDH leads to ROS formation in mitochondria by coupling proline oxidation to reduction of the respiratory electron transport chain [30,57]. Indeed, the highest activation of *ProDH* (on 10 days after drought treatment) coincided with the most accumulated proline and ROS level in an ABA-dependent manner (Figure 1B and Figure 4B,F). Mani et al. [60] reported that altered levels of *ProDH* cause hypersensitivity to proline and its analog. Moreover, an exceptional enhancement of *P5CDH*, the second enzyme for proline catabolism, was observed on day 10 following Exo-H_2_O_2_ treatment, which showed a considerable accumulation (68.2 nmol g^−1^ FW) of endogenous H_2_O_2_ (Figure 1B). Thus, an overexpression of *ProDH* and *P5CDH* might be part of the hypertensive response to over-produce H_2_O_2_ and/or proline, predicting an increase in proline-P5C cycling, leading to ROS accumulation [25,57,61].

## 5. Conclusions

**Figure 6:**
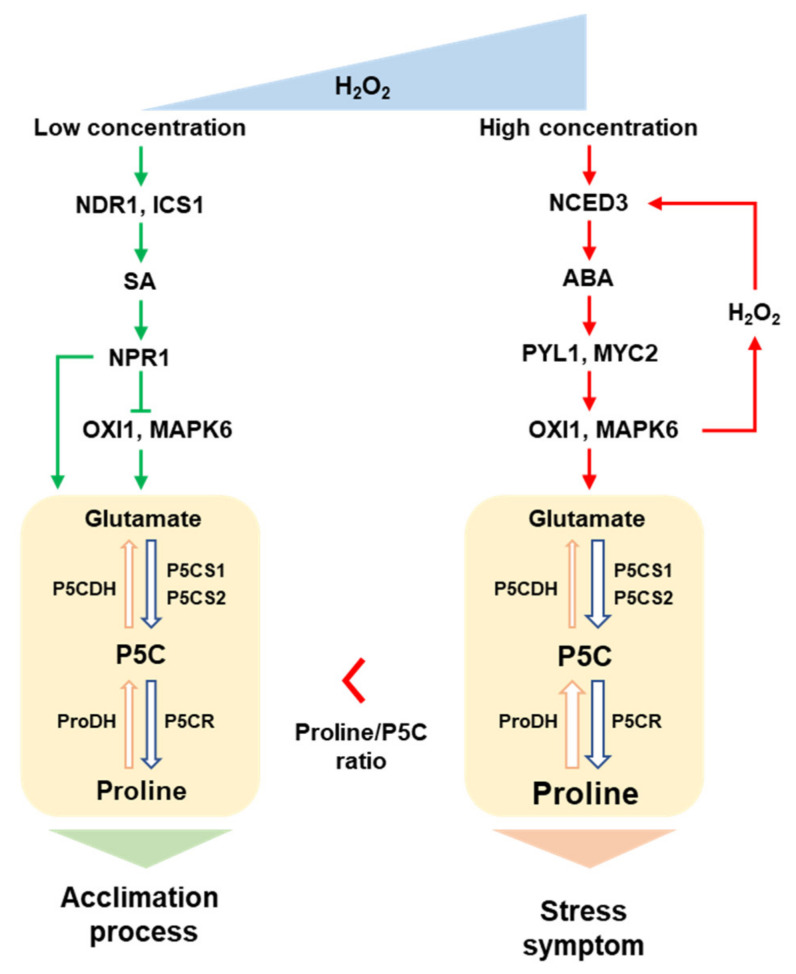
Proposed model of crosstalk between ROS signaling, hormones, and proline metabolism in response to endogenous H_2_O_2_ level. Green and red arrows represent the SA- and ABA-dependent pathways, respectively.

The present data indicate that hormonal interaction with proline metabolism is governed by endogenous levels of ROS (especially H_2_O_2_), as shown by SA-mediated activation of proline synthesis at lower endogenous H_2_O_2_ levels, and a predominant ABA-dependent proline accumulation along with ROS accumulation. Furthermore, to the best of our knowledge, the present data are the first to report that H_2_O_2_-responsive SA and ABA involves in ROS signaling and proline metabolism in rapeseed leaves (Figure 6), representing two distinct phases characterized by the following: (1) an active NDR1-mediated SA-dependent proline synthesis with upregulation of *P5CS* and *P5CR*, and depression of *ProDH* as an acclamatory process at lower level of endogenous H_2_O_2_ produced by either Exo-H_2_O_2_ or drought treatment; and (2) drought-induced proline accumulation with ABA-dependent MAPK6 activation via *OXI1*, leading to upregulation of *ProDH* as hypersensitive responses to a higher H_2_O_2_ level. Future studies are necessary (1) to define the threshold at which proline level switches from inducing cellular protection to hypersensitivity to over-produced ROS and/or proline, and (2) to elucidate hormonal interaction with proline in redox regulation.

## Figures and Tables

**Table 1:** The changes in leaf water potential (*Ψ*_w_), chlorophyll content, and lipid peroxidation level under control, exogenous H_2_O_2_ (Exo-H_2_O_2_), or drought treatments for 10 days.

	Days after Treatment		
	0	5	10
Leaf water potential (*Ψ*_w_, MPa)			
Control	−0.44 ± 0.02 ^a^	−0.44 ± 0.02 ^a^	−0.48 ± 0.02 ^a^
Drought		−0.66 ± 0.03 ^b^	−1.20 ± 0.02 ^d^
Exo-H_2_ O_2_		−0.47 ± 0.02 ^a^	−1.07 ± 0.04 ^c^
Chlorophyll (mg g^−1^ FW)			
Control	1.61 ± 0.08 ^ab^	1.73 ± 0.10 ^a^	1.62 ± 0.09 ^ab^
Drought		1.45 ± 0.07 ^abc^	1.22 ± 0.06 ^c^
Exo-H_2_ O_2_		1.63 ± 0.09 ^ab^	1.34 ± 0.05 ^bc^
Lipid peroxidation (MDA, nmol g^−1^ FW)			
Control	4.55 ± 0.24 ^d^	4.46 ± 0.32 ^d^	4.84 ± 0.23 ^cd^
Drought		5.85 ± 0.24 ^bc^	9.45 ± 0.33 ^a^
Exo-H_2_O_2_		4.80 ± 0.35 ^cd^	6.33 ± 0.43 ^b^

Results are represented as mean ± SE for *n* = 3. Different letters in a vertical column or a horizontal row indicate values that are significantly different at *p* < 0.05 according to Duncan’s multiple range test.

**Table 2:** The changes in levels of endogenous abscisic acid (ABA) and salicylic acid (SA), and in the ratio of ABA to SA under control, drought, or exogenous H_2_O_2_ (Exo-H_2_O_2_) treatments for 10 days.

	Days after Treatment		
	0	5	10
ABA (ng g^−1^ FW)			
Control	5.24 ± 0.29 ^c^	6.44 ± 0.36 ^c^	5.93 ± 0.11 ^c^
Drought		32.66 ± 0.04 ^b^	115.09 ± 5.56 ^a^
Exo-H_2_O_2_		7.24 ± 0.14 ^c^	33.45 ± 0.34 ^b^
SA (ng g^−1^ FW)			
Control	2.21 ± 0.20 ^f^	2.69 ± 0.36 ^ef^	3.21 ± 0.11 ^e^
Drought		7.09 ± 0.13 ^b^	4.18 ± 0.19 ^d^
Exo-H_2_O_2_		13.45 ± 0.37 ^a^	5.49 ± 0.24 ^c^
ABA/SA ratio			
Control	2.40 ± 0.13 ^d^	2.48 ± 0.20 ^d^	1.85 ± 0.03 ^d^
Drought		4.61 ± 0.08 ^c^	27.55 ± 0.74 ^a^
Exo-H_2_O_2_		0.54 ± 0.01 ^e^	6.12 ± 0.23 ^b^

Results are represented as mean ± SE for *n* = 3. Different letters in a vertical column or a horizontal row indicate values that are significantly different at *p* < 0.05 according to Duncan’s multiple range test.

## Data Availability

Data is contained within the article and its Supplementary Materials.

## Supplementary Materials

The following supporting information can be downloaded at: www.mdpi.com/article/10.3390/antiox11030566/s1, Supplementary Table S1: Primer sequences used for qRT-PCR analysis.
