# Supplementary material for: H2O2-Responsive Hormonal Status Involves Oxidative Burst Signaling and Proline Metabolism in Rapeseed Leaves
Source: Antioxidants (Basel). 2022 Mar 16;11(3):566. doi: 10.3390/antiox11030566 (PMC8944793; doi:10.3390/antiox11030566)
Supplement: Supplementary file 1 [file antioxidants-11-00566-s001.zip › antioxidants-1618642-supplementary.pdf]

Supplementary Materials

Table S1. Specific primers used for qRT-PCR.

Target gene	GenBank Number	Forward sequence (5´-3´)	Reverse sequence (5´-3´)
BnNCED3	HQ260434	GGAGTGCTTCTGCTTCCATC	TTCGAGGTTGACTTGCTCCT
BnPYL1	XM_009132867	ACCGCAGACCTACAAGCACT	CTCTCCCTCTCGAACCTGTG
BnMYC2	XM013880351	ACCAAACGTCTCGAAAATGG	TGTCAACGAGCAAGAGGATG
BnICS1	XM013887885	TCAATCCCAGAACGAGATCC	GACAGAAACCTTCGGATGGA
BnNDR1	XM_022695985	CCCTCTTTCTCCTCCAATCC	TTCTCCTTGAACGCAGAGGT
BnNPR1	EF613226.1	TGAGAACATTGCCAAGCAAG	CAACAGCAAAATGGAGAGCA
BnNADPH oxidase	XM_013847449	CACCTCTCCCTCTTTCTGT	CGTTGGGGTTTTGTCGCTAT
BnMAPK6	XM_013884849.2	GCTAGCTCCATGGGACAGAG	GAGCAGTTGGTGGTGGATTT
BnOXI1	XM_013843315.2	GCCACCAACTACCACAGGAT	CCCAAGCAATGACAAAACCT
BnP5CS1	AF314811	CGATTTGGACTTGGTGCTGA	GCCCATCCTCTCCTAGTC
BnP5CS2	AF314812	CCATTATCTTCCTCCTCTCAC	AACAACTGCTGTCCCAACC
BnP5CR	XM013812259	TTCAGTAATGAGCCTTGGAA	TCTGTGAAGCTAAACCCAAA
BnProDH	EU375567	CGATTTGGACTTGGTGCTGA	GCCCATCCTCTCCTAGTC
BnP5CDH	XM013787440	CTACGAACTCGTCACCAAAG	AGGTCCAAACCAATGATTCT
BnActin	AF111812.1	GATTCCGTTGCCCTGAAGTA	GCGACCACCTTGATCTTCAT
